# Isoforskolin and forskolin attenuate lipopolysaccharide‐induced inflammation through TLR4/MyD88/NF‐κB cascades in human mononuclear leukocytes

**DOI:** 10.1002/ptr.6248

**Published:** 2019-01-14

**Authors:** Xiaohua Du, Rui Shi, Youlan Wang, Wenjuang Wu, Shibo Sun, Zelan Dai, Chen Chen, Zhiying Weng, Xian Li, Qian Liu, Liyan Zhang, Mayer Saidian, Weimin Yang

**Affiliations:** ^1^ School of Pharmaceutical Science and Yunnan Key Laboratory of Pharmacology for Natural Products Kunming Medical University Kunming China; ^2^ First Affiliated Hospital of Kunming Medical University Kunming China; ^3^ Kunming Medical Science Research Institute Kunming China; ^4^ Second Affiliated Hospital of Kunming Medical University Kunming China; ^5^ Beckman Laser Institute, University of California Irvine California; ^6^ The Institute for Drug Research, School of Pharmacy Hebrew University of Jerusalem Jerusalem Israel

**Keywords:** FSK, inflammation, ISOF, MyD88, NF‐κB, TLR4

## Abstract

The principal active component of isoforskolin (ISOF) is from the plant *Coleus forskohlii*, native to China, which has attracted much attention for its biological effects. We hypothesize that ISOF and forskolin (FSK) pretreatment attenuates inflammation induced by lipopolysaccharide (LPS) related to toll‐like receptor 4 (TLR4), myeloid differentiation factor 88 (MyD88), and nuclear factor kappa B (NF‐κB) signaling. Mononuclear leukocytes (MLs) from healthy donors' blood samples were separated by using density gradient centrifugation. Protein levels of TLR4, MyD88, and NF‐κB were detected using western blot and inflammatory cytokines interleukin (IL) 1β, IL‐2, IL‐6, IL‐21, IL‐23, tumor necrosis factor (TNF) α, and TNF‐β were tested by enzyme‐linked immunosorbent assay and Quantibody array in MLs. Our results showed that LPS augmented the protein levels of TLR4, MyD88, and NF‐κB in MLs and the production of IL‐1β, IL‐2, IL‐6, IL‐21, IL‐23, TNF‐α, and TNF‐β in supernatants of MLs. Despite treatment with ISOF and FSK prior to LPS, the protein levels of TLR4, MyD88, NF‐κB, IL‐1β, IL‐2, IL‐6, IL‐21, IL‐23, TNF‐α, and TNF‐β in MLs were apparently decreased. roflumilast (RF) and dexamethasone (DM) had a similar effect on MLs with ISOF and FSK. Our results, for the first time, have shown that ISOF and FSK attenuate inflammation in MLs induced by LPS through down‐regulating protein levels of IL‐1β and TNF‐α, in which TLR4/MyD88/NF‐κB signal pathway could be involved.

## 1 INTRODUCTION

The plant *Coleus forskohlii* is distributed primarily in India, Thailand, China, Egypt, and Brazil and has a history of use in the treatment of multiple diseases. Isoforskolin (ISOF), also named 6‐acetyl‐7‐deacetyl forskolin, was isolated from *C*. *forskohlii*, a tropical perennial plant native to Yunnan in China (Yang et al., 2011). It is reported that the Yunnan native plant does not mainly contain forskolin (FSK) but ISOF, which has attracted attention for its biological functions. Our previous study demonstrated that ISOF activated adenylyl cyclases (ACs) and then increased cyclic adenosine monophosphate (cAMP) levels in rat liver homogenate, and it also relaxed the contraction of isolated guinea pig lung and trachea smooth muscle induced by histamine (Wang & Cao, 2013). Moreover, pretreatment with ISOF attenuated acute lung injury (ALI) in animal models and decreased the production of inflammatory cytokines tumor necrosis factor (TNF) α, interleukin (IL) 1β, IL‐6, and IL‐8 (Yang et al., 2011). One of our members' research indicated that ISOF down‐regulated the transcription and expression of TNF‐α and IL‐6 in murine macrophages, human macrophages, and dendritic cells induced by recombinant *Borrelia burgdorferi* basic membrane protein A and may have a potential clinical application as an anti‐inflammatory agent for the treatment of Lyme arthritis (Zhao et al., 2017).

ISOF is an analog of diterpene FSK, ISOF is extracted from *C*. *forskohlii* distributed in Yunnan, China, and FSK is extracted from *C*. *forskohlii* distributed in India. It is well known that FSK is an effective AC activator and causes an increase in intracellular cAMP. Many researches showed that cAMP‐induced modulation of the receptor for advanced glycosylation end products isoform in macrophages can control the inflammatory state in both in vitro and in vivo experimental conditions (Motoyoshi et al., 2014). FSK has effects on anti‐inflammation through cAMP signaling (Sousa et al., 2010; You, Xiong, Zhang, Shi, & Shi, 2017). Our previous study also showed ISOF activated ACs, increased cAMP, and then attenuated inflammation in ALI induced by lipopolysaccharide (LPS; Yang et al., 2011). Furthermore, another study showed AC activation attenuated transmembrane toll‐like receptor 4 (TLR4) signaling in a murine macrophage cell line (RAW264.7) and bone marrow‐derived macrophages when stimulated with LPS (Cai, Du, Feng, et al., 2013). Therefore, we hypothesized that ISOF and FSK activated ACs and then regulated TLR4 signaling.

TLR4 mediates the inflammatory process evoked by LPS (Hu, Lou, Mao, et al., 2016). Myeloid differentiation factor 88 (MyD88) is a toll‐like receptor (TLR) adaptor. After recognition by TLR4, a series of cascades including MyD88 are initiated, and then MyD88 primarily activates nuclear factor kappa B (NF‐κB) family members and mitogen‐activated protein kinase (Liu & Ding, 2016). Activation of NF‐κB has a variety of biological functions such as control DNA transcription, cytokine production and cell survival time, and extensive involvement in the body's immune system, inflammation, and stress physiological pathology (Baker, Hayden, & Ghosh, 2011). Therefore, inhibition of NF‐κB activity is an effective way to block inflammation.

On the basis of the above factors and to better understand biological effects of ISOF, we hypothesized that ISOF and FSK pretreatment attenuated inflammatory reaction through down‐regulation inflammatory factors such as the protein levels of TNF‐α, IL‐1β, and other inflammatory factors in supernatant of mononuclear leukocytes (MLs) induced by LPS, and the mechanism of anti‐inflammation of ISOF partly related to TLR4/MyD88/NF‐κB signal pathway.

## 2 MATERIALS AND METHODS

### 2.1 Chemicals and kits

ISOF and FSK (purity 99%) were purified as white crystal by Kunming Baker Norton Pharmaceutical Co., Ltd. DM injection was purchased from Yangtze River Pharmaceutical Company (Yangzhuo, Zhejian, China). LPS (Escherichia coli 055:B5),

dimethyl sulfoxide (DMSO; 0227), trypan blue powder (72571), and roflumilast (SML1099) were purchased from Sigma‐Aldrich Co. (St Louis, MO). Sterile water for injection (H41024923) was purchased from Sinopharm Group Health and Pharmaceutical Co. (Tianjin, China). Protein chip assay kit (QAH‐TH17‐1) was purchased from RayBiotech, Inc. (Guangzhou, China). Enzyme‐linked immunosorbent assay (ELISA) kit for IL‐1β (VAL 101, the minimum detectable dose of IL‐1β is typically less than 1.0 pg/ml) and TNF‐α (VAL105, the minimum detectable dose of TNF‐α is typically less than 7.8 pg/ml) were purchased from R&D Systems, Inc. (Minneapolis, MN). Mouse TLR4 polyclonal antibody (ab13556), rabbit MYD88 polyclonal antibody (ab2068), rabbit NF‐κB (P65) antibody (ab7970), and anti‐beta actin antibody (ab49900) were purchased from Abcam Company (USA). Rabbit GAPDH monoclonal antibody (2118) was purchased from Cell‐signaling Technology Inc (Massachusetts, USA). Lymphocyte separation solution (LTS1007N) and Human Blood preservation solution were purchased from Tianjin Haoyang Biological Products Technology Co., Ltd (Tianjin, China).

### 2.2 Protocols in human peripheral blood mononuclear cells

Human peripheral blood mononuclear cell samples from healthy volunteers were kindly supplied by the First Affiliated Hospital of Kunming Medical University. The hospital ethics committee approved the study and all the volunteers provided informed consent. Human MLs were isolated by using density gradient centrifuge (Fuss, Kanof, Smith, & Zola, 2009; Kanof, Smith, & Zola, 2001). The procedures yield a 95–98% viable MLs (by trypan dye exclusion) and 95% pure MLs population (by morphology in Giemsa stains). Cells were washed by phosphate‐buffered saline buffer and resuspended in Roswell Park Memorial Institute 1640 medium containing 1% bovine serum albumin, and cell count was adjusted to 5 × 10^6^/ml for use. MLs were suspended in 6‐well plates (2 ml/well); preincubated with at 37°C (5% CO_2_) for 2 hr; given pretreatment with ISOF (1.0 μM), FSK (0.5 μM), RF (0.5 μM), DM (25 μM), and vehicle control (DMSO) for 30 min at 37°C; and then challenged with LPS (1 μg/ml) for 6 hr at 37°C. At the end of the incubation period, cell supernatants were collected and stored at −20°C for measurements of TNF‐α and IL‐1β. MLs were collected for measurements of TLR4, MyD88, and NF‐κB by using western blot method.

### 2.3 Quantification of cytokine analysis in supernatants of MLs

To investigate the cytokine expression of MLs treated with ISOF, FSK, RF, and DM, cytokines were analyzed by using Quantibody array in supernatants of MLs (Quantibody Human TH17 Array 1: granulocyte‐macrophage colony‐stimulating factor [GM‐CSF], IL‐1β, IL‐2, IL‐4, IL‐5, IL‐6, IL‐10, IL‐12p70, IL‐13, IL‐17A, IL‐17F, IL‐21, IL‐22, IL‐23, interferon [IFN] **γ**, CCL20, transforming growth factor [TGF] β1, TNF‐α, and TNF‐β; RayBiotech, Guangzhou, China), according to the manufacturer's specifications. Each sample was prepared in quadruplicate. InnoScan 300 Microarray Scanner software was used to collect fluorescence intensities. Detection limits for cytokines are displayed on the manufacturer's website (RayBiotech). Protein levels of ML supernatants were analyzed by RayBiotech antibody chip and then identified by ELISA.

### 2.4 Measurement of TNF‐α and IL‐1β by ELISA

Protein levels of TNF‐α and IL‐1β in ML supernatants were assayed using a commercially available ELISA kit (human TNF‐α and IL‐1β ELISA, R&D, USA). A polyclonal antibody specific for TNF‐a and IL‐1β was used for coating the 96‐well microtiter plates. Briefly, 100 ml of the different standards was added into the appropriate wells in duplicate. One hundred milliliter of supernatants was added in duplicate into additional wells. The plate was covered and incubated on the constant temperature culture shaker for 2 hr at 25°C. After extensive washing to remove unbound compounds, TNF‐α and IL‐1β was recognized by the addition of 100 ml of polyclonal antibody specific for TNF‐α and IL‐1β (detection antibody), and then the plate was covered and incubated on the constant temperature culture shaker for 1.5 hr at 25°C. After removal of excess polyclonal antibody and repeated washing to remove unbound compounds, 100 ml of horseradish peroxidase‐conjugated antirabbit IgG (secondary antibody conjugate) was added, and the plate was covered and incubated on the constant temperature culture shaker for 0.5 hr at 25°C. Subsequently, the bound peroxidase activity was quantified using 50 ml of the substrate 3,39,5,59‐tetramethyl benzidine. The color reaction was developed at ambient temperature in the dark for 10 min. The intensity of the color reaction was measured at 450 nm after acidification and was directly proportional to the concentration of TNF‐α and IL‐1β in the standards. According to a standard curve established using recombinant TNF‐α and IL‐1β, the concentration of TNF‐α and IL‐1β in individual samples were calculated. The minimum detectable dose of IL‐1β is typically less than 1.0 pg/ml. The minimum detectable dose of TNF‐α is typically less than 7.8 pg/ml.

### 2.5 Western blot analysis

The TLR4, MyD88, and NF‐κB protein levels in lysed cell were examined by western blot analysis. Protein concentrations were determined by using a BCA Protein Assay Kit (Beyotime Biotechnology, Haimen, Jiangsu, China). Total protein (20 μg) was fractionated on 10% sodium dodecyl sulfate–polyacrylamide gel electrophoresis and transferred to nitrocellulose membranes. Membranes were blocked with 10% defatted milk powder solution at room temperature for 2 hr and incubated overnight at 4°C with the rabbit antibodies against TLR4 (concentrate on 1:500), MyD88 (concentration 1:700), NF‐κB (concentration 1:700), GAPDH (concentration 1:10,000), and beta‐actin (concentration 1:2,000). After three washes, membranes were incubated with horseradish peroxidase‐conjugated goat antirabbit IgG antibody for 1.5 hr at room temperature. Then, washing was repeated four times, and the protein was visualized with an enhanced chemiluminescence kit (Sigma, USA). The density values of bands were quantified by densitometric analysis of scanned images (Scion Image 4.03). The relative protein ratio was calculated by determining the integrated intensity of the bands of each treated group as a ratio of the control condition.

### 2.6 Statistical analysis and calculations

Data was expressed as means ± *SEM*. Statistical analysis was performed using statistical software Sigma Stat 3.5. Comparisons were made using one‐way analysis of variance, followed by Student‐Newman‐Keuls (SNK) test analysis if there are normal distribution and variance homogeneity of data; one‐way analysis of variance on rank was used if there is no homogeneity of data variance. *p* < 0.05 was considered statistically significant. The graph was performed using software Sigma plot10.

Cytokines of ML supernatants detected by Quantibody Human TH17 Array 1 were scanned by using InnoScan 300 Microarray Scanner and were analyzed by using Quantibody Human TH17 Array Data analysis software.

## 3 RESULTS

### 3.1 Effects of ISOF/FSK on cytokine expression in ML supernatants by cytokine array analysis

**Figure 1:**
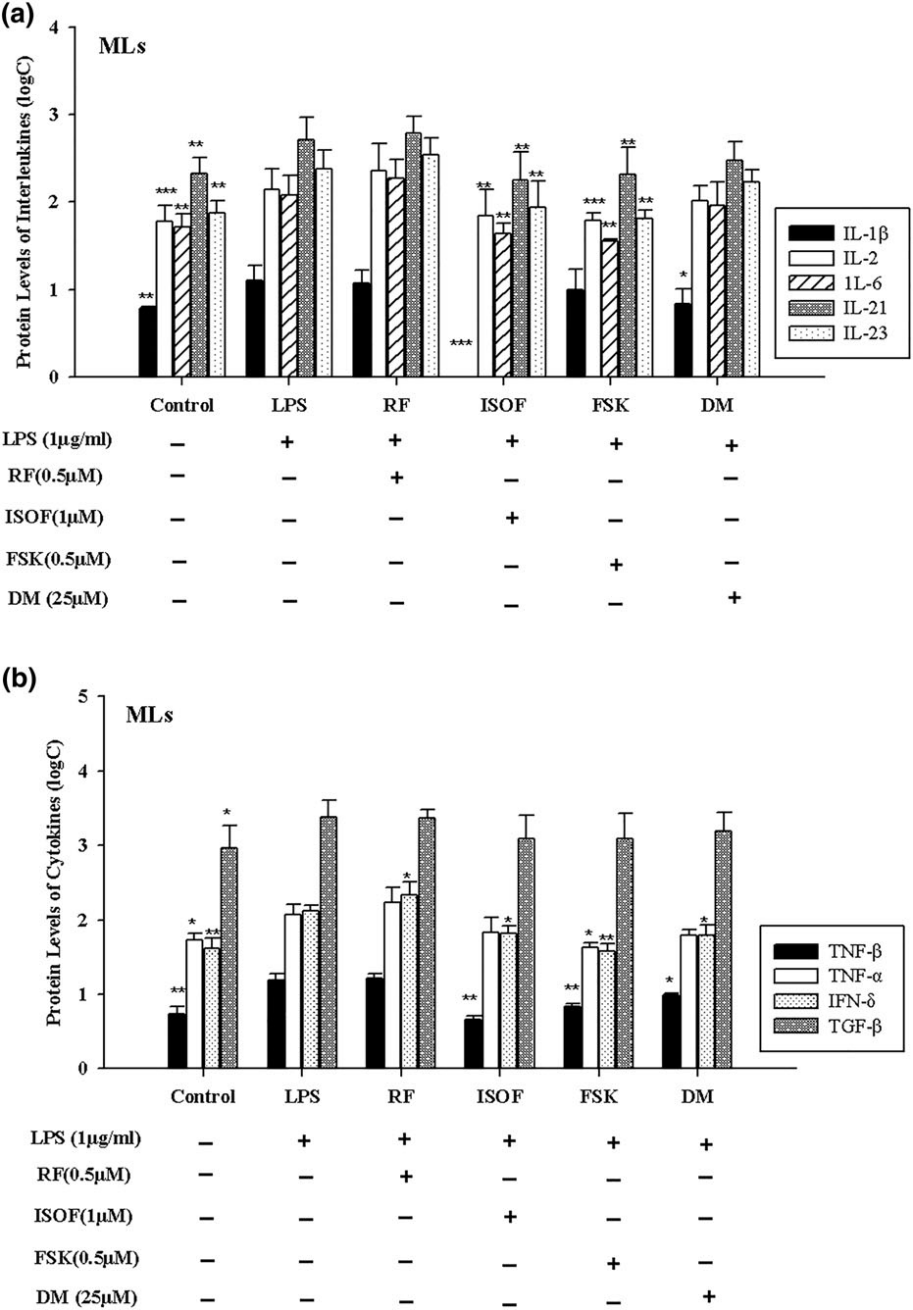
Effects of isoforskolin (ISOF) on protein levels of interleukin and cytokines in MLs supernatants. (a) Effect of ISOF on protein levels of interleukin in MLs supernatants. (b) Effect of ISOF on protein level of cytokines in mononuclear leukocytes (MLs) supernatants. Control group, MLs were only given solvent (dimethyl sulfoxide [DMSO] volume concentration was 1/1,000) without lipopolysaccharide (LPS). LPS, ISOF, forskolin (FSK), RF and DM groups were given pre‐treatment with DMSO, ISOF (0.5 μM), ISOF (1.0 μM), FSK (0.5 μM), RF (0.5 μM), DM (25 μM) respectively for half an hour prior to LPS (1 μg/ml) treatment for 6 hr. MLs supernatants were collected for detection of cytokines. Log C is derived from raw data (pg/L) through logarithmic transformation. interleukin (IL)–1β, IL‐2, IL‐6, IL‐21, IL‐23, and tumor necrosis factor (TNF)–β were analyzed by one‐way analysis of variance (ANOVA), whereas TNF‐α and transforming growth factor‐β1 were analyze by one‐way ANOVA on rank. ^*^ *p* < 0.05, ^**^ *p* < 0.01, ^***^ *p* < 0.001, compared with LPS group. Data are means ± *SEM*; 𝑛 = 4 repeat experiments with the same volunteer

All 20 cytokines were detected in control, LPS, ISOF, FSK, RF, and DM groups. As seen from Table 1, some GM‐CSF, IL‐5, IL‐10, IL‐13, and TNF‐β were expressed in a few, and some IL‐4, IL‐12p70, IL‐17, IL‐17F, IL‐22, IL‐24, IL‐28α, and macrophage inflammatory protein‐3α were not detected at all. Compared with control group, LPS treatment increased protein expression of IL‐1β, IL‐2, IL‐6, IL‐21, IL‐23, TNF‐α, and TNF‐β in supernatants of MLs. After being given treatments with ISOF (1.0 μM), FSK (0.5 μM), RF (0.5 μM), and DM (25 μM) prior to LPS treatment, the effects of LPS on increase in protein levels of IL‐1β, IL‐2, IL‐6, IL‐21, IL‐23, TNF‐α, and TNF‐β were inhibited (Table 1, Figure 1a,b). Moreover, we also found that most protein levels of cytokines were higher in the ISOF (0.5 μM) group than in the ISOF (1.0 μM) group. Compared with ISOF (1.0 μM), ISOF (0.5 μM) had a weak effect on down‐regulation of IL‐1β, IL‐2, IL‐6, IL‐21, IL‐23, TNF‐α, and TNF‐β in MLs treated with LPS.

**Table 1:** Effects of ISOF and other positive control drugs on cytokines in the supernatants of MLs

(pg/ml)	Control	LPS1.0	RF0.5	ISOF0.5	ISOF1.0	FSK0.5	DM25
GM‐CSF	0.0 ± 0.0	12.6 ± 0.3	39.9 ± 2.4	29.4 ± 0.6	0.0 ± 0.0	0.9 ± 0.05	4.1 ± 0.1
IFN‐γ	41.6 ± 1.8^**^	132.1 ± 2.3	216.1 ± 8.1^*^	201.4 ± 3.5	65.2 ± 1.8^*^	38.4 ± 2.2^**^	61.3 ± 1.4^*^
IL‐1β	6.1 ± 0.1^**^	12.6 ± 1.0	11.9 ± 0.8	9.1 ± 0.1	0.0 ± 0.0^***^	9.9 ± 1.2	6.8 ± 0.7^*^
IL‐2	59.8 ± 3.1^***^	138.0 ± 7.7	227.3 ± 15.0	218.1 ± 5.3	69.2 ± 5.7^**^	61.4 ± 1.4^***^	103.9 ± 4.4
IL‐4	0.0 ± 0.0	0.0 ± 0.0	7.7 ± 0.4	4.3 ± 0.1	0.0 ± 0.0	0.0 ± 0.0	0.0 ± 0.0
IL‐5	4.0 ± 0.2	9.7 ± 0.3	13.6 ± 0.3	13.3 ± 0.1	1.2 ± 0.1	1.7 ± 0.0	4.7 ± 0.4
IL‐6	52.0 ± 2.2^**^	118.4 ± 6.4	186.6 ± 8.6	174.5 ± 5.1	44.0 ± 1.5^**^	36.0 ± 0.2^**^	92.2 ± 6.3
IL‐10	1.7 ± 0.0	2.5 ± 0.1	4.3 ± 0.2	3.2 ± 0.1	0.8 ± 0.0	0.9 ± 0.0	1.6 ± 0.1
IL‐12p70	0.0 ± 0.0	0.7 ± 0.0	3.9 ± 0.0	2.6 ± 0.0	0.0 ± 0.0	0.0 ± 0.0	0.1 ± 0.0
IL‐13	3.2 ± 0.2	15.7 ± 0.7	20.5 ± 0.6	22.9 ± 0.8	7.0 ± 0.1	2.3 ± 0.2	8.0 ± 0.6
IL‐17	2.3 ± 0.1	0.3 ± 0.0	0.7 ± 0.1	3.1 ± 0.0	0.0 ± 0.0	0.0 ± 0.0	0.2 ± 0.0
IL‐17F	0.0 ± 0.0	0.0 ± 0.0	0.0 ± 0.0	0.0 ± 0.0	0.0 ± 0.0	0.0 ± 0.0	0.0 ± 0.0
IL‐21	211.4 ± 8.3^**^	518.6 ± 24.4	603.7 ± 21.2	749.5 ± 22.1	177.7 ± 12.5^**^	206.1 ± 13.7^**^	300.2 ± 13.0
IL‐22	0.0 ± 0.0	0.0 ± 0.0	0.0 ± 0.0	0.0 ± 0.0	0.0 ± 0.0	0.0 ± 0.0	0.0 ± 0.0
IL‐23	74.9 ± 2.8^**^	235.9 ± 10.9	345.2 ± 13.4	361.7 ± 14.0	87.0 ± 6.8^**^	65.0 ± 1.6^**^	168.0 ± 5.4
IL‐28A	1.1 ± 0.0	0.0 ± 0.0	0.0 ± 0.0	0.0 ± 0.0	1.9 ± 0.0	0.0 ± 0.0	0.0 ± 0.0
MIP‐3α	0.9 ± 0.0	0.0 ± 0.0	0.0 ± 0.0	0.0 ± 0.0	0.0 ± 0.0	0.0 ± 0.0	0.0 ± 0.0
TGF‐β1	923.0 ± 47.0^*^	2,402.6 ± 78.5	2,360.8 ± 36.0	2,107.6 ± 32.5	1,237.3 ± 61.5	1,224.9 ± 66.7	1,537.6 ± 60.3
TNF‐α	53.1 ± 1.5^*^	116.1 ± 7.0	171.1 ± 4.9	167.0 ± 4.3	67.1 ± 3.9	42.8 ± 0.8^*^	62.7 ± 1.3
TNF‐β	5.50 ± 0.3^**^	15.30 ± 0.6	16.6 ± 0.3	18.9 ± 0.2	4.60 ± 0.1^**^	6.8 ± 0.2^**^	9.70 ± 0.1^*^

*Note*. FSK: forskolin; GM‐CSF: granulocyte‐macrophage colony‐stimulating factor; IFN: interferon; IL: interleukin; ISOF: isoforskolin; LPS: lipopolysaccharide; MIP: macrophage inflammatory protein; ML: mononuclear leukocyte; TGF: transforming growth factor; TNF: tumor necrosis factor. Zero indicates the value is lower than the lowest values of the standard curve that can be detected. In the control group, MLs were only given solvent (DMSO volume concentration was 1/1000) without LPS. LPS, ISOF, FSK, RF, and DM groups were given pretreatment with DMSO, ISOF (0.5 μM), ISOF (1.0 μM), FSK (0.5 μM), RF (0.5 μM), and DM (25 μM), respectively for half an hour, and then each group was given a treatment with LPS (1 μg/ml) for 6 hr. The ML supernatants were collected for detection of cytokines. Data are means ± *SEM*; 𝑛=4 repeat experiments with the same volunteer. IL‐1β, IL‐2, IL‐6, IL‐21, IL‐23, and TNF‐β were analyzed by one‐way analysis of variance (ANOVA), whereas TNF‐α and TNF‐β1 were analyze by one‐way ANOVA on rank.

* *p* < 0.05.

** *p* < 0.01.

*** *p* < 0.001 compared with LPS group.

### 3.2 Effects of ISOF/FSK on TNF‐α and IL‐1β in human ML supernatants by ELISA assay

**Figure 2:**
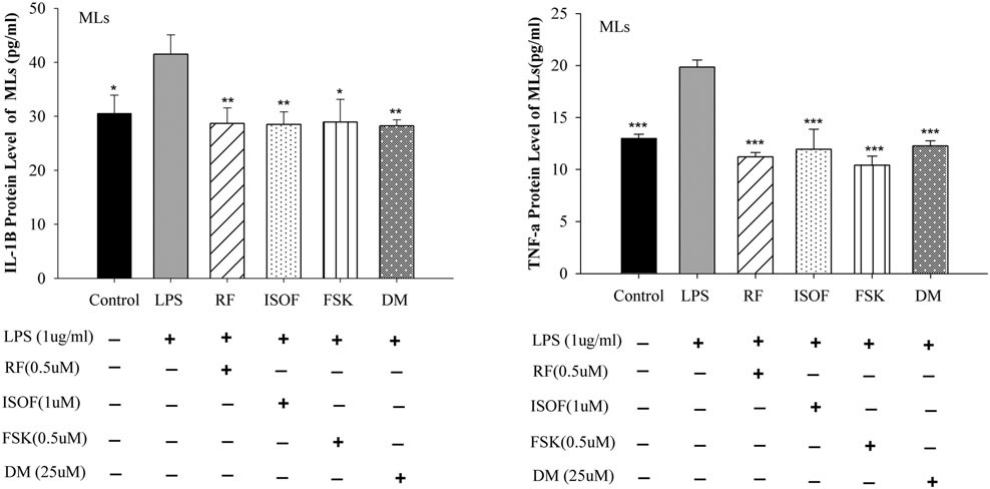
Effects of isoforskolin (ISOF) on tumor necrosis factor (TNF)–α and interleukin (IL)–1β protein levels in supernatants of mononuclear leukocytes (MLs). (a) Effect of ISOF and other control drugs pre‐treatment on TNF‐α protein expression in MLs supernatant. (b) Effect of ISOF and other control drugs pre‐treatment on IL‐1β protein expression in ML supernatant. TNF‐α and IL‐1β (6 hr after LPS challenge) production in the MLs supernatants were evaluated by enzyme‐linked immunosorbent assay method, respectively. In control group, MLs were only given solvent (dimethyl sulfoxide volume concentration was 1/1,000) without lipopolysaccharide (LPS). LPS, ISOF, forskolin (FSK), RF, and DM groups were given pretreatment with DMSO, ISOF (1.0 μM), FSK (0.5 μM), RF (0.5 μM), DM (25 μM), respectively for 0.5 h, and then each group was given treatment with LPS (1 μg/ml) for 6 hr. One‐way analysis of variance followed by SNK was used to process the data of TNF‐α and IL‐1β. ^*^ *p* < 0.05, **p < 0.01, and^***^ *p* < 0.001, compared with LPS group. Data are means ± *SEM*; 𝑛 = 5 independent experiments with independent volunteer

In order to verify the results of quantification of cytokine analysis, TNF‐α and IL‐1β in ML supernatants were tested by the ELISA method. As seen from Figure 2a,b, compared with the control group, LPS augmented the protein levels of TNF‐α and IL‐1β in ML supernatants, whereas when given pretreatment with ISOF, FSK, RF, and DM, the pro‐inflammatory effect of LPS on MLs was apparently inhibited. Compared with the LPS group, with the pretreatment with ISOF (1.0 μM), FSK (0.5 μM), RF (0.5 μM), and DM (25 μM), the protein levels of TNF‐α and IL‐1β of MLs were attenuated sharply.

### 3.3 Effects of ISOF/FSK on TLR4, MyD88, and NF‐κB in human MLs

TLR4 is a transmembrane protein, member of the TLR family, which belongs to the pattern recognition receptor family. Its activation can activate MyD88 and can then lead to an intracellular signaling pathway NF‐κB and inflammatory cytokine production.

**Figure 3:**
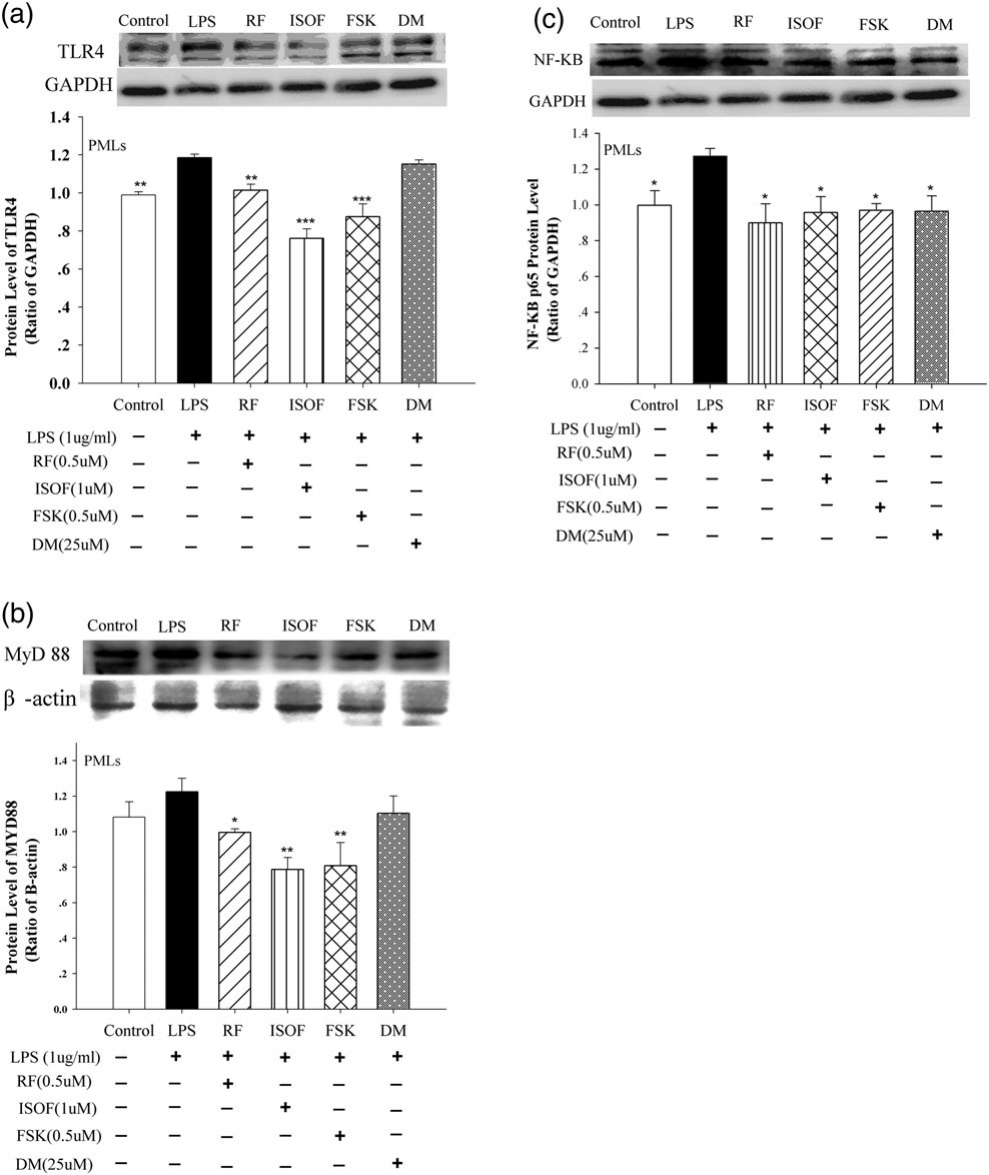
Effects of isoforskolin (ISOF) and forskolin (FSK) on toll‐like receptor 4 (TLR4), myeloid differentiation factor 88 (MyD88), and nuclear factor kappa B (NF‐κB) protein level of mononuclear leukocytes (MLs). (a) Representative immunoblot and quantification of TLR4 protein expression in MLs with ISOF and other control drugs pre‐treatment. (b) Representative immunoblot and quantification of MyD88 protein expression in MLs with ISOF and other control drugs pretreatment. (c) Representative immunoblot and quantification of NF‐κB protein expression in MLs with ISOF and other control drugs pre‐treatment. Protein ratio of control was calculated by determining band integrated intensity as ratio of control. Control group, MLs were only given solvent (dimethyl sulfoxide [DMSO] volume concentration was 1/1,000) without lipopolysaccharide (LPS). LPS, ISOF, FSK, RF and DM groups were given pre‐treatment with DMSO, ISOF (1.0 μM), FSK (0.5 μM), RF (0.5 μM), DM (25 μM) respectively for 0.5 h, and then each group was given treatment with LPS (1 μg/ml) for 6 hr. One‐way analysis of variance (ANOVA) followed by SNK was used to process the data of TLR4 and NF‐κB. One‐way ANOVA on rank followed by SNK was used to process the data of MyD88. ^*^ *p* < 0.05, ^**^ *p* < 0.01, ^***^ *p* < 0.001, compared with LPS group. Data are means ± *SEM*; 𝑛 = 5 independent experiments with independent volunteer

The content of key molecules of TLR4 pathway was quantified by western blot. As seen in Figure 3a,b, compared with the control group, LPS treatment increased protein levels of TLR4 and MyD88 in human MLs. With the treatment with ISOF (1.0 μM), FSK (0.5 μM), RF (0.5 μM), and DM (25 μM) prior to LPS, compared with LPS used alone, protein levels of TLR4 and MyD88 were reduced in MLs. Particularly, there were apparently decreases in protein levels of TLR4 and MyD88 in ISOF, FSK, and RF groups.

As seen from Figure 3c, compared with control group, LPS treatment increased protein level of NF‐κB p65 in MLs. After MLs were treated with ISOF (1.0 μM), FSK (0.5 μM), RF (0.5 μM), and DM (25 μM) prior to LPS, compared with LPS used alone, protein levels of NF‐κB p65 in ISOF, FSK, RF, and DM groups were severely decreased.

## 4 DISCUSSION

ISOF was isolated from *C*. *forskohlii*, native to Yunnan, China. Except for our group, few have been reported regarding the biological activity of ISOF due to the source of *C*. *forskohlii*. Our result is the first to show that ISOF, to our knowledge, can attenuate inflammation in MLs induced by LPS through down‐regulating protein expression of IL‐1β, IL‐2, IL‐6, IL‐21, IL‐23, TNF‐α, TNF‐β, TLR4, MyD88, and NF‐κB; and the molecular mechanism was partly related to the TLR4/MyD88/NF‐κB signal pathway. Moreover, FSK also has a similar effect on inflammation in MLs induced by LPS.

Our result shows that ISOF attenuates inflammation in MLs induced by LPS through down‐regulating protein expression of inflammatory factors. Furthermore, ISOF has the following effects: suppressing ocular hypertension in rabbits (Li et al., 2000); increasing cAMP levels in rat liver homogenate; relaxing the contraction of isolated guinea pig lung and trachea smooth muscle induced by histamine (Wang & Cao, 2013); attenuating ALI in animal models induced by LPS; down‐regulating pro‐inflammatory cytokines TNF‐α, IL‐1β, IL‐6, and IL‐8 (Yang et al., 2011); inhibiting the transcription and expression of TNF‐α and IL‐6 in murine macrophages, human macrophages, and dendritic cells induced by recombinant *B*. *burgdorferi* basic membrane protein A (Zhao et al., 2017); and reducing cough and sputum as well as inhibiting airway remodeling and inflammation by regulating the expression of TGF‐β1 and IL‐1β in rat asthma model (Liang et al., 2017). These data indicate that ISOF can be a novel drug for inflammation, especially in airway and lung. And in our other research about cell toxicity of ISOF where human small airway epithelial cells (BEAS‐2B) were cultured with ISOF (2.5, 5, 10, 20, and 50 μM) for 6, 12, and 24 hr, the results showed that ISOF under 2.5 μM of concentration had no cytotoxicity (manuscript under review).

ISOF, chemically named as 6‐acetyl‐7‐deacetyl FSK, is a principal component of *C*. *forskohlii* Briq. native to Yunnan, China, whereas FSK is the principal ingredient of Indian C. *forskohlii*. Our data shows that both ISOF and FSK inhibit inflammatory response in MLs induced by LPS, but the relationship between them is not known and need to be further studied.

Numerous researches show that FSK is an effective AC activator and causes an increase in intracellular cAMP and then plays the effects of anti‐inflammation (Sousa et al., 2010; You et al., 2017). This study is similar with our results. Moreover, FSK has effects of weight loss (Doseyici, Mehmetoglu, Toker, Yerlikaya, & Erbay, 2014), decreasing fasting blood glucose levels (Rios‐Silva, Trujillo, Trujillo‐Hernandez, et al., 2014), reducing intraocular pressure in patient with glaucoma (Vetrugno et al., 2012), nerve protection (Jensen, Ducray, Widmer, & Meyer, 2015), improving depression (Menninger & Tabakoff, 1997), antitumor (Follin‐Arbelet et al., 2015), antiaging (Tumer, Bowman, Larochelle, Kelley, & Scarpace, 1997), and dilating bronchi (Kanne, Burte, Prasanna, & Gujjula, 2015).

LPS is the major stimulus for the release of inflammatory mediators (Pittet, Mackersie, Martin, & Matthay, 1997). Administration of LPS to experimental cells causes the pathological condition of ongoing sepsis and concomitant ALI/acute respiratory distress syndrome (ARDS)‐like systemic inflammatory reaction; therefore, we selected LPS to stimulate human MLs, which mainly contain a variety of cells involved in inflammatory response and immune regulation, such as lymphocyte, MLs, and macrophages, as causing the pathological condition of the ongoing sepsis.

As the receptor of LPS, TLR4 plays a principal role in the recognition of gram‐negative bacteria. MyD88 is an adaptor molecule in response to the interaction of TLR4 with LPS (Xiang et al., 2015). In the present study, our results showed that LPS treatment increased the protein levels of TLR4, MyD88, and NF‐κB in human MLs. This result was similar with previous research (He et al., 2016) and further demonstrated that LPS signaled via TLR4 and MyD88 activated NF‐κB in anti‐inflammatory reaction. And our previous study also showed that ISOF can attenuate inflammation in BEAS‐2B induced by LPS through down‐regulating protein expression of TLR4, MyD88, and NF‐κB (this manuscript is under review now). In addition, our research illustrated protein levels of NF‐κB could be apparently raised in MLs stimulated by LPS. These results were the same as numerous research studies in the past (Lin, Chen, Chen, Chang, & Chou, 2015). NF‐κB activation is central to the development of pulmonary inflammation and ALI. Our research also showed ISOF could counteract the effect of LPS on increasing protein levels of NF‐κB p65 in MLs, it indicated ISOF had anti‐inflammation through decreasing the NF‐Κb p65 expression.

Other than NF‐κB p65, other pro‐inflammatory cytokines such as IL‐1β, IL‐2, IL‐6, IL‐8, and TNF‐a were important mediators of the inflammatory response in ALI/ARDS (Mokra & Kosutova, 2015), In ALI/ARDS, IL‐1β, TNF‐α, IL‐8, and IL‐6, which are usually increased, in the bronchoalveolar lavage fluid of patients at risk of ARDS or with established ARDS, IL‐1β, TNF‐a (Park, Goodman, Steinberg, et al., 2001), and IL‐8 (Gonzalez‐Lopez, Garcia‐Prieto, Batalla‐Solis, et al., 2012) were elevated. Additionally, high concentrations of IL‐6 may serve as a predictive marker of poor outcome (Bouros, Alexandrakis, Antoniou, et al., 2004). Our detecting results of quantification of cytokine analysis chip showed that the protein levels of IL‐1β, IL‐2, IL‐6, IL‐21, IL‐23, TNF‐α, and TNF‐β in the LPS group was higher than the control group in supernatants of MLs, but pretreatment with ISOF, FSK, and DM, respectively, showed that the effects of LPS on MLs were inhibited. This indicated that the ISOF, FSK, and DM have anti‐inflammation effect on MLs induced by LPS through adjusting the expression of IL‐1β, IL‐2, IL‐6, IL‐21, IL‐23, TNF‐a, and TNF‐β, but that some cytokines needed to be further identified by experiments.

In the present study, except for the RF group, the change trend of protein levels of TNF‐α and IL‐1β detected by ELISA and quantification of cytokine analysis were similar in other experimental groups. The value of cytokines detected by quantification of cytokine analysis chip in the RF group were higher than that in the LPS group, the reason might be that the dose of RF may have been too high for that person.

In addition, we also found that DM was able to attenuate expression of inflammation cytokines NF‐κB p65, TNF‐α, and IL‐1β in MLs induced by LPS, but it could not effectively reduce the protein level of TLR4 and MyD88. The reasons may be that DM had anti‐inflammation effect on MLs induced by LPS related to another pathway, but TLR4/MyD88 pathway.

In conclusion, the results of this study revealed that ISOF and FSK attenuate inflammation in MLs induced by LPS through down‐regulating protein levels of pro‐inflammatory cytokines IL‐1β, IL‐2, IL‐6, IL‐21, IL‐23, TNF‐α, and TNF‐β, in which TLR4/MyD88/NF‐κB signal pathway could be involved. These findings suggest that ISOF and FSK could be a potential candidate for the treatment of inflammation induced by LPS.

## FUNDING INFORMATION

This work was partially supported by the National Natural Science Foundation of China (81870037, 81173110, 81560589, 81402991, 81860012), Yunnan Provincial Science and Technology Department (2017FA043, 2014BC012, 2014IA033,2017FE467‐028, 2017FE467‐019, 2018FE001‐026, and 2017IC041), and the Yunnan Provincial Education Department (ZD2015009 and 2015C009Y).

## CONFLICTS OF INTEREST

The funders had no role in the study design, data collection and analysis, decision to publish, or preparation of the manuscript. The authors state no conflict of interest.
